# The plasma kynurenine-to-tryptophan ratio as a biomarker of tuberculosis disease in people living with HIV on antiretroviral therapy: an exploratory nested case–control study

**DOI:** 10.1186/s12879-024-09258-4

**Published:** 2024-04-02

**Authors:** Sivaporn Gatechompol, René Lutter, Frédéric M. Vaz, Sasiwimol Ubolyam, Anchalee Avihingsanon, Stephen J. Kerr, Frank van Leth, Frank Cobelens

**Affiliations:** 1 HIV-NAT, Thai Red Cross AIDS Research Centre, Bangkok, Thailand; 2 Center of Excellence in Tuberculosis, Faculty of Medicine, Chulalongkorn University, Bangkok, Thailand; 3 Department of Global Health and Amsterdam Institute for Global Health and Development, Amsterdam University Medical Centers Location University of Amsterdam, Amsterdam, The Netherlands; 4 Department of Experimental Immunology, Amsterdam UMC and Amsterdam Infection and Immunity Institute, University of Amsterdam, Amsterdam, The Netherlands; 5 Department of Pulmonary Diseases, Amsterdam UMC, University of Amsterdam and VU University Amsterdam, Amsterdam, The Netherlands; 6 Department of Core Facility Metabolomics, Amsterdam UMC, Amsterdam, The Netherlands; 7 Laboratory Genetic Metabolic Diseases, Department of Clinical Chemistry and Pediatrics, Amsterdam Gastroenterology Endocrinology Metabolism, Amsterdam UMC, University of Amsterdam, Amsterdam, The Netherlands; 8 Biostatistics Excellence Centre, Faculty of Medicine, Chulalongkorn University, Bangkok, Thailand; 9 Department of Health Sciences, Vrije Universiteit Amsterdam, Amsterdam Public Health Research Institute, Amsterdam, The Netherlands

**Keywords:** IDO, Tuberculosis, HIV, Diagnostic, Monitoring

## Abstract

**Background:**

Non-sputum-based tests are needed to predict or diagnose tuberculosis (TB) disease in people living with HIV (PWH). The enzyme indoleamine 2, 3-dioxygenase-1 (IDO1) is expressed in tuberculoid granuloma and catabolizes tryptophan (Trp) to kynurenine (Kyn). IDO1 activity compromises innate and adaptive immune responses, promoting mycobacterial survival. The plasma Kyn-to-Trp (K/T) ratio is a potential TB diagnostic and/or predictive biomarker in PWH on long-term antiretroviral therapy (ART).

**Methods:**

We compared plasma K/T ratios in samples from PWH, who were followed up prospectively and developed TB disease after ART initiation. Controls were matched for age and duration of ART. Kyn and Trp were measured at 3 timepoints; at TB diagnosis, 6 months before TB diagnosis and 6 months after TB diagnosis, using ultra performance liquid chromatography combined with mass spectrometry.

**Results:**

The K/T ratios were higher for patients with TB disease at time of diagnosis (median, 0.086; IQR, 0.069–0.123) compared to controls (0.055; IQR 0.045–0.064; p = 0.006), but not before or after TB diagnosis. K/T ratios significantly declined after successful TB treatment, but increased upon treatment failure. The K/T ratios showed a parabolic correlation with CD4 cell counts in participants with TB (*p* = 0.005), but there was no correlation in controls.

**Conclusions:**

The plasma K/T ratio helped identify TB disease and may serve as an adjunctive biomarker for for monitoring TB treatment in PWH. Validation studies to ascertain these findings and evaluate the optimum cut-off for diagnosis of TB disease in PWH should be undertaken in well-designed prospective cohorts.

**Trial registration:**

ClinicalTrials.gov Identifier: NCT00411983.

**Supplementary Information:**

The online version contains supplementary material available at 10.1186/s12879-024-09258-4.

## Background

In the past decades a variety of diagnostic tests for tuberculosis (TB) ranging from smear microscopy to nucleic acid amplification test have become available. Despite this, TB morbidity and mortality are still unacceptably high. In 2021, there were an estimated 1.4 million deaths among HIV-negative people and an additional 187,000 deaths among people living with HIV (PWH) [1]. Among PWH, paucibacillary and extra-pulmonary TB (EPTB) pose diagnostic challenges [2, 3]. Diagnosis of EPTB requires a high degree of clinical suspicion and special diagnostic procedures in order to get microbiologically positive specimens, while PWH with TB less often produce sputum [4, 5]. Therefore, sensitive and specific non-sputum-based tests are needed to predict the development of clinical TB, so rapid diagnostic and management decisions can be made.

Indoleamine 2, 3-dioxygenase (IDO)1 is an inducible tryptophan-catabolizing enzyme expressed in tuberculoid granuloma [6]. IDO1 like its isoform IDO2, which is enzymatically less active and rarely expressed, and the constitutively expressed liver enzyme tryptophan dioxygenase (TDO) catabolize tryptophan (Trp) to kynurenine (Kyn) [7]. The production of kynurenine is the rate-limiting step in the IDO/TDO-kynurenine pathway, thus changes in kynurenine are reflect alterations in the IDO1 activity. IDO1 activity can therefore be estimated using the Kyn-to-Trp ratio in plasma (K/T ratio). IDO-mediated Trp depletion results in T-cell anergy and apoptosis, whereas the accumulation of Kyn suppresses T-cell differentiation and function [8]. Animal studies have demonstrated that *M. tuberculosis* infection is associated with high expression of IDO in the granuloma, leading to compromised T cell function which promotes mycobacterial survival and growth [9, 10].

IDO activity reflected by the K/T ratio has been proposed as a potential biomarker for the diagnosis of TB, and monitoring treatment outcomes in children [11], pregnant women [12], HIV-negative [13, 14] and HIV-positive [15] individuals. This enzyme has also been recognized as an immune response biomarker that plays a pivotal role in HIV immune dysfunction [7]. A previous longitudinal PWH cohort demonstrated that plasma K/T ratios were elevated 6 months before clinical TB symptoms became apparent, and declined after successful treatment [15]. A recent study in pregnant women living with HIV demonstrated plasma K/T ratios were elevated in women with TB disease at time of diagnosis [12]. However, no studies yet have evaluated the performance of the K/T ratio as a TB diagnostic or predictive biomarker among PWH on long-term antiretroviral therapy (ART). In this study, we evaluated whether the plasma K/T ratio could predict the occurrence of TB disease, diagnose TB disease, and monitor treatment response in PWH.

## Methods

### Study participants

This study used biobanked plasma samples from HIV-NAT 006 (HN006), a prospective, clinic-based cohort that has enrolled adults living with HIV aged ≥ 18 years since 1996 (Clinicaltrials.gov NCT00411983, first registered on 15/12/2006). In this cohort, the participants were seen every 6 months at HIV-NAT, Thai Red Cross AIDS Research Centre, Bangkok. Care for HIV infection was provided according to the prevailing Thai treatment guidelines at the time participants attended follow-up visits. The following variables were collected at each clinic visit: body temperature, body weight, body mass index (BMI), physical examination findings, full differential blood counts, lipid profile, creatinine, and alanine aminotransferase (ALT). CD4 + and CD8 + lymphocyte counts performed by automated fluorescence-activated flow cytometry as well as HIV RNA were measured every 12 months.

Plasma samples were collected and stored at each visit. Ten mL of whole blood were collected in ethylene diamine tetra acetate (EDTA) tubes and centrifuged (3000 rpm) at 4ºC for 20 min. Plasma samples were aliquoted into volumes of 500 μL and stored at ≤ -70ºC until analysis.

### Ethical approval

The study protocol was approved by the medical ethics committee, Faculty of Medicine, Chulalongkorn University, Bangkok (IRB No.161/45) and conducted in accordance with Good Clinical Practice guidelines and the principles of the Declaration of Helsinki. All participants in HN006 cohort provided written informed consent and consented for the use of plasma stored samples in this study.

### Definition of case and control participants

During the follow-up visits, participants with symptoms and/or signs of TB disease were assessed by physical examination, chest radiography, sputum smear microscopy and culture for *M. tuberculosis* and/or GeneXpert MTB/RIF (Xpert; Cepheid, Sunnyvale, CA, which was available from 2012 onward). TB disease was bacteriologically confirmed by smear microscopy, culture or Xpert, or clinically diagnosed based on radiologic evidence of TB without bacteriological confirmation combined with a good clinical response to antituberculosis treatment.

**Fig. 1:**
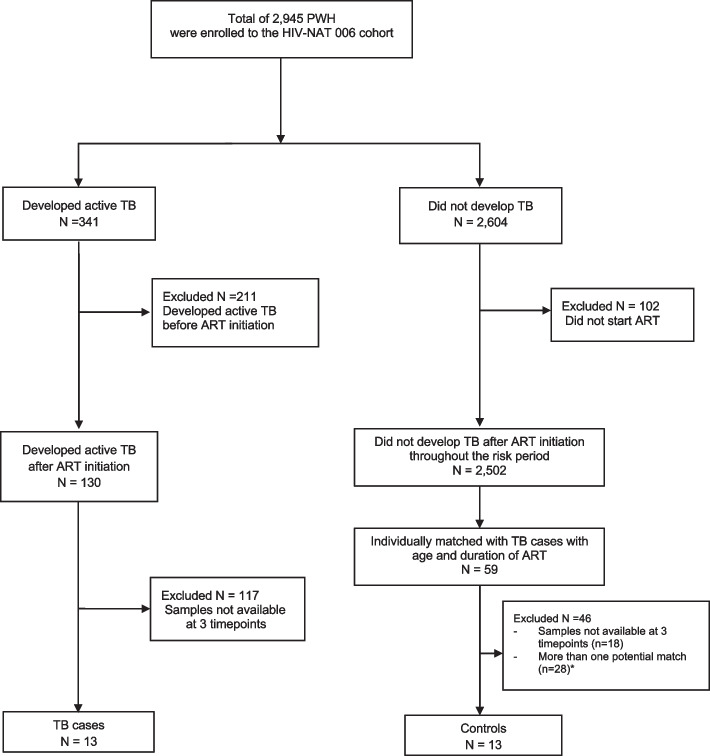
The study flow chart describing participants recruitment ^*^Six cases had only one match; 7 cases had one or more potential matches. For these cases, the control was randomly selected from available matches

We included participants with TB disease who had samples available at 3 different time points: 1) 6 months before TB diagnosis, 2) around TB diagnosis (the allowable window for sample collection was 2 months before or after initiation of TB treatment), and 3) 6 months after TB diagnosis. We matched controls from HN006 cohort with individual cases (1:1) based on age and ART duration with one year window around the date of the TB diagnosis visit. Then, we further selected only controls that with samples available at all 3 timepoints. If there was more than one control eligible to be matched with individual cases, the control was randomly selected. We did not test for latent TB infection in the controls, since TB preventive therapy was not recommended by Thai National guidelines at that time. According to the clinical records, control participants had no respiratory symptoms or signs and symptoms of active TB at visit 2, (the visit used to match with TB cases. The study flow chart describing participant recruitment is shown in Fig. 1.

#### Quantitation of plasma tryptophan, kynurenine and downstream metabolites

Kyn and Trp were separated by ultra-performance liquid chromatography and measured by tandem mass spectrometry (UPLC-MS/MS) system (Waters, Milford, MA, USA). The selected plasma samples were shipped at or below -70 °C to the Amsterdam University Medical Centers for analysis. Upon analyses, samples were thawed rapidly at room temperature and a mixture of stable isotope-labeled internal standards was added to 50 μl of plasma. The samples were deproteinized using acetonitrile, dried under nitrogen, and reconstituted in 100 μl of 0.1% heptafluorobutyric acid. Aliquots (10 μl) of the extracts were injected into the UPLC–MS/MS system comprising of Acquity Xevo TQ-XS system operated in positive ESI mode using multiple reaction monitoring (MRM) for preselected analytes and an overall run time was 6 min. The MRM transition which gave the most intense signal was chosen for quantification. IDO activity was calculated as the ratio of measured kynurenine concentration to measured tryptophan concentration (K/T ratio). In order to capture all metabolites in the Trp and Kyn pathways, we measured other metabolites including anthranilic acid, 3OH-kynurenine, 3OH-anthranilic acid, quinolinic acid, kynurenic acid and xanthurenic acid (Supplementary Fig. 1). We also calculated the ratio of the sum of all determined kynurenine metabolites to Trp concentration (Sum/T ratio).

### Statistical Analysis

Non-normally distributed data was presented as medians with interquartile ranges (IQR). Cross-sectional comparisons of plasma Trp concentration, Kyn concentration, and the K/T ratio between TB patient and control samples at each visit were tested using the Mann–Whitney U test. Changes of K/T ratio between the visits among TB patients were tested using Wilcoxon signed-rank test. Based on two-way scatterplot appearances, regression models with quadratic terms were used to investigate associations between the K/T ratio at TB diagnosis visit and clinical variables including CD4 count and body mass index (BMI). Pearson’s correlation coefficient was calculated from the predicted regression values.

We calculated a receiver operating characteristic curve (ROC) to evaluate the most optimal cutoff point for K/T ratio that can be used to diagnose TB based on Liu’s index. We assessed the sensitivity, specificity and discriminative ability of the model using the area under the ROC curve (AROC) and 95% CIs around these estimates were obtained by bootstrapping the estimates with 1000 replication.

A *P*-value for two-sided test less than or equal to 0.05 was considered statistically significant. The statistical analysis was done by using Stata version 17 (StataCorp., College Station, TX, USA) and figures were done by using GraphPad Prism 9 software (GraphPad Software, USA). 

## Results

### Demographic data of the study participants

Among 130 participants who developed active TB after ART initiation (Fig. 1), we identified 13 pulmonary TB patients with three consecutive plasma samples available. Ten were bacteriologically confirmed and 3 were clinically diagnosed. At TB diagnosis visit, the clinical characteristics of TB patients included fever (85%), weight loss (62%), cough (46%) and cervical lymphadenopathy (39%). At the time of TB diagnosis, three (23%) TB cases had detectable HIV viral load. The HIV viral loads for these cases were 5.75, 6.29 and 6.41 log10 copies/mL, respectively. All controls had undetectable HIV viral load (< 40 copies/ml).

Ten TB patients were successfully treated with the standard TB 6-month regimen (isoniazid, rifampin, pyrazinamide, and ethambutol). Three patients had poor treatment response due to poor adherence and adverse effects from TB treatment.

The median age of the TB patients at TB diagnosis was 41.6 years (interquartile range (IQR), 36.1–52.1). All of the participants were Thai and 8/13 (62%) were male. The median CD4 cell count at TB diagnosis was 370 (189–577) cells/mm^3^ and median duration of ART was 12.2 (4.2–16.8) years. Of the samples taken at the time of TB diagnosis among the TB patients, 9/13 (69.2%) were collected at a median of 1 (0.8–1.7) month before TB treatment initiation while 4/13 (30.8%) were collected at a median of 0.9 (0.8–1) month after TB treatment initiation. The demographic and clinical characteristics of the patients with TB and the controls are summarized in Table 1.

**Table 1:** Demographic and clinical characteristics of the study participants with HIV

Clinical characteristics^a^	TB patients (*N*=13)	Controls (*N*=13)
**Age (years), median (IQR)**	41.6 (36.1-52.1)	47.4 (36.9-51.8)
**Males, N (%)**	8 (62)	9 (69)
**BMI (kg/m**^**2**^**), median (IQR)**	19.9 (18.8-21.6)	23.4 (21.4-24.4)
**CD4 cell count (cells/mm**^**3**^**), median (IQR)**	370 (189-577)	560 (486-730)
**Duration of ART (years), median (IQR)**	12.2 (4.2-16.8)	13.1 (7.7-14.6)

^a^Clinical characteristics were measured for TB patients at TB diagnosis and for the controls at visit 2

*ART* Antiretroviral therapy, *BMI* Body mass index, *N* number, *IQR* interquartile range

#### Plasma kynurenine and tryptophan ratio among PWH with tuberculosis and controls

**Fig. 2:**
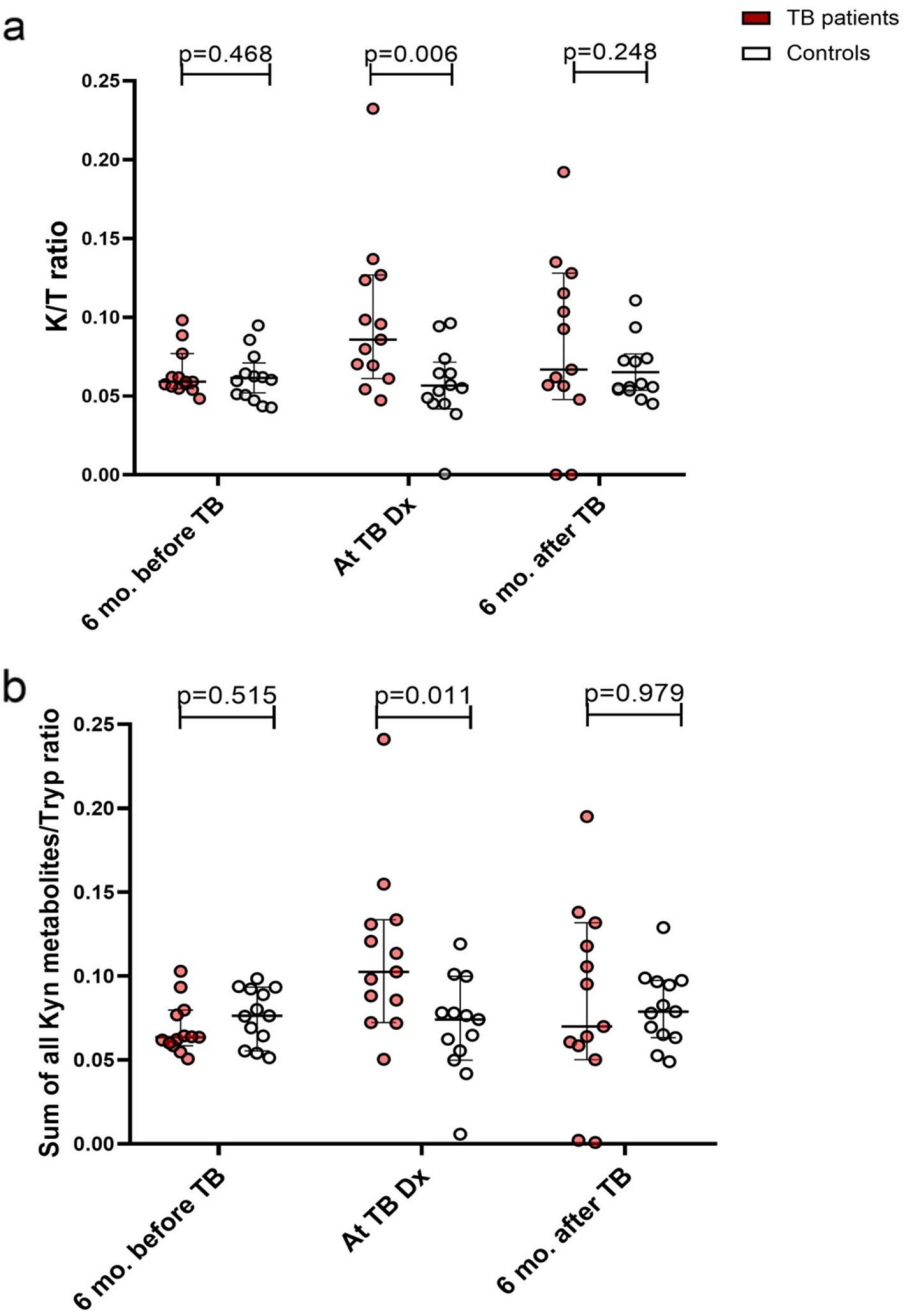
**a** Plasma kynurenine to tryptophan ratio and **b** sum of metabolites to tryptophan ratio in TB participants compared to that in controls at each visit. The bars represent the median with error bars indicating 95% confidence interval. The *p* values were calculated using Mann–Whitney test

**Fig. 3:**
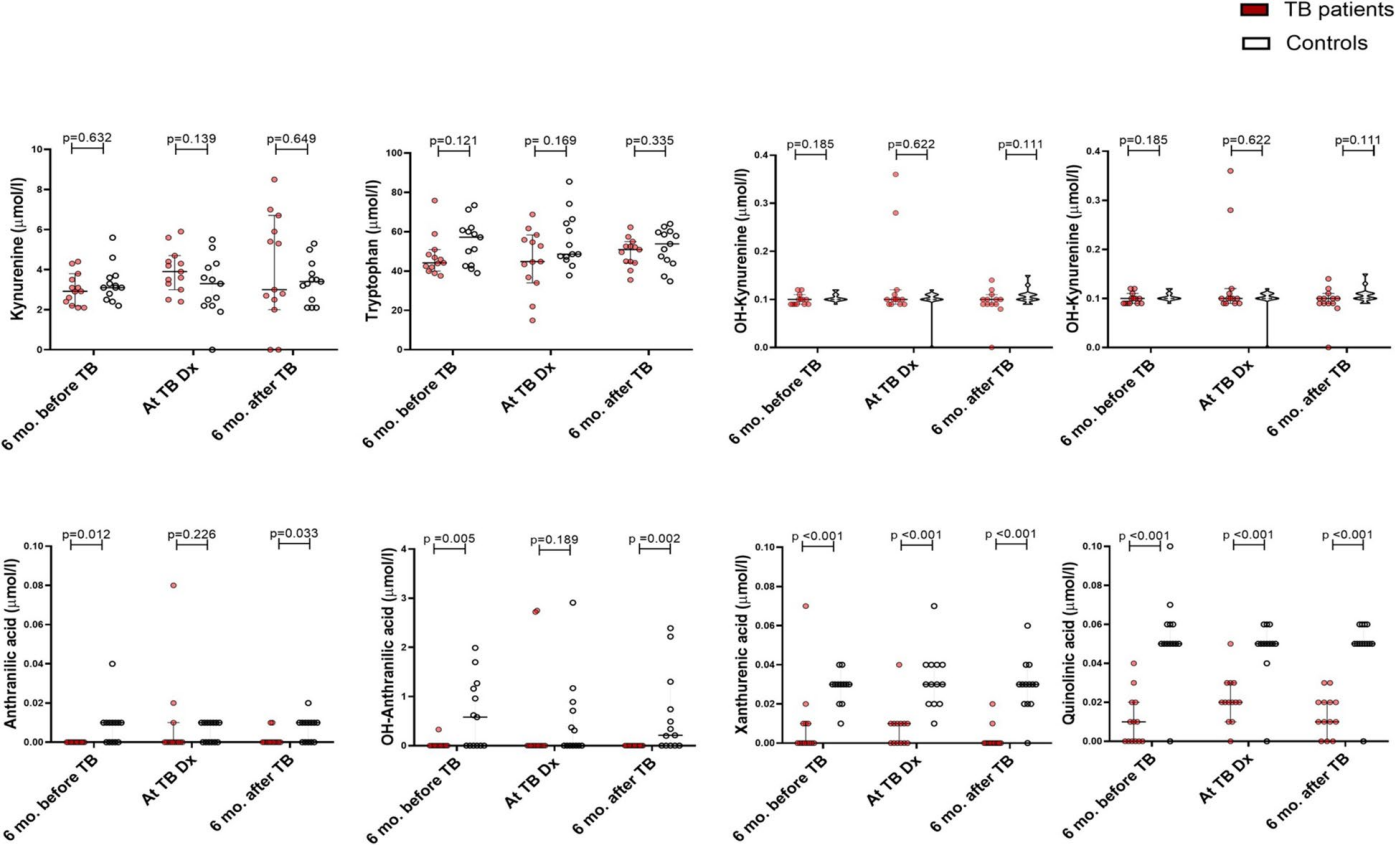
Concentrations of tryptophan and kynurenine metabolites in plasma from TB patients and controls at each visit. The bars represent the median with error bars indicating 95% confidence interval. The Y axis scale differs between the metabolites

At the time of TB diagnosis, the K/T ratio was significantly higher among participants with TB (median, 0.086 (0.069–0.123)) compared to the controls at the corresponding visit (0.055 (0.045–0.064); *p* = 0.006). There were no significant differences between study groups at the visits 6 months before or after TB diagnosis (Fig. 2a). The sum of the metabolites to tryptophan ratio was also significantly higher at TB diagnosis (Fig. 2b). Assessing each metabolite in the pathway separately, we found no significant differences in the concentration of kynurenine, tryptophan and OH-kynurenine between TB participants and controls. However, concentrations of anthranilic acid, kynurenic acid, OH-anthranilic acid, xanthurenic acid and quinolinic acid were significantly lower in the participants with TB compared to the controls (Fig. 3), including timepoints 6 months before and 6 months after TB diagnosis.

In addition, we conducted a sensitivity analysis which excluded the 3 TB participants with poor treatment outcomes. This sensitivity analysis gave similar results: at the time of TB diagnosis, the K/T ratio was significantly higher among participants with TB (median, 0.097; IQR, 0.070–0.127) compared to the controls at the corresponding visit (0.055; IQR 0.045–0.064; *p* = 0.004) (Fig. S2).

### The plasma K/T ratio was associated with clinical response after TB treatment

**Fig. 4:**
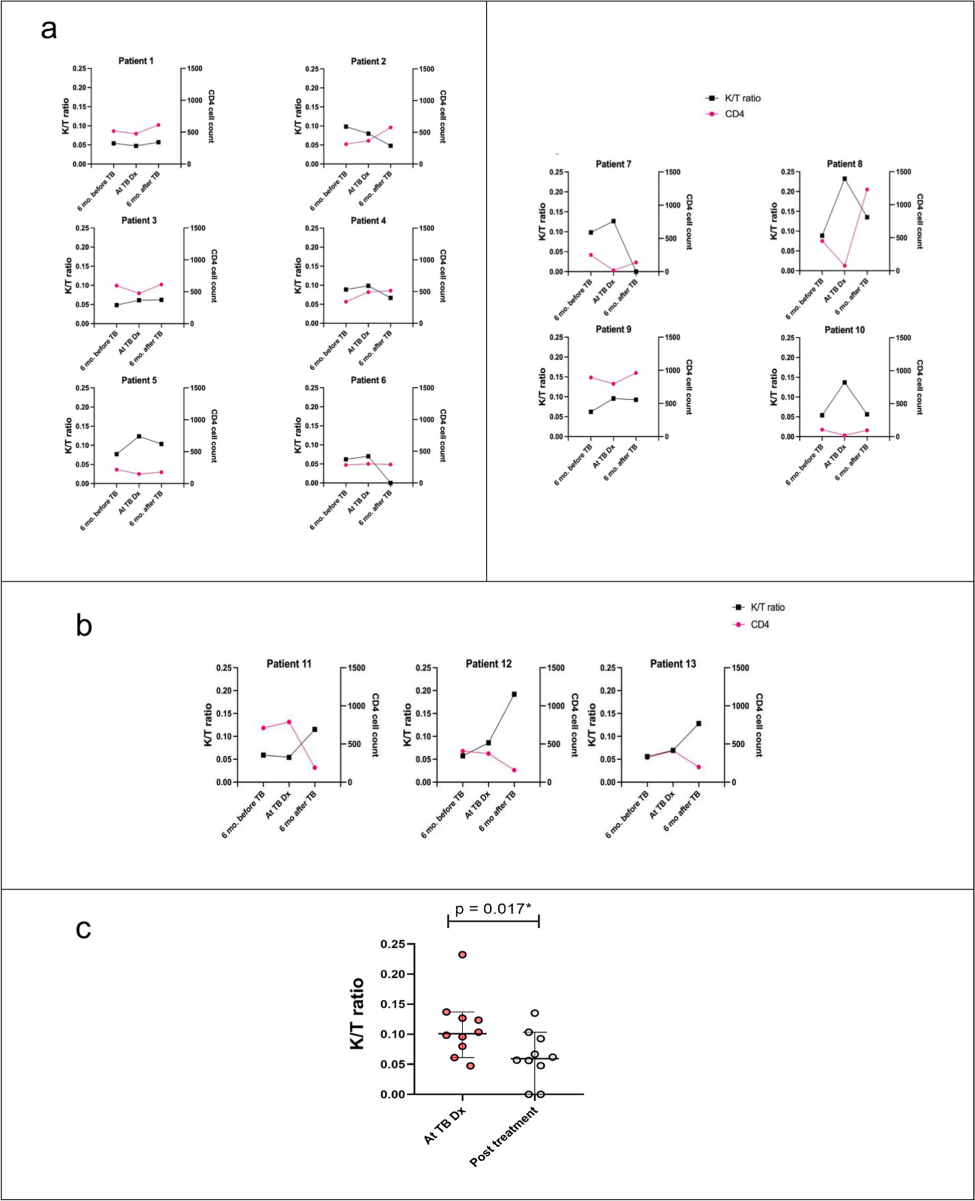
**a** The plasma K/T ratio after TB treatment in participants who were treated successfully. **b** The plasma K/T ratio and CD4 count of the participants who had treatment failure. Each dot or line represents an individual. **c** The median of K/T ratio at the time of TB diagnosis compared to 6 months post treatment among TB participants who were treated successfully

The majority of participants who were treated successfully had significantly decreased K/T ratios after TB treatment (Fig. 4a). For these, at the time of TB diagnosis, the median K/T ratio was 0.064 (0.049–0.096) compared to 0.057 ((0.053–0.734), p = 0.017) 6 months post treatment (Fig. 4c). In contrast, the three participants who experienced treatment failure had higher levels of K/T ratio as well as lower levels of CD4 blood cell counts after TB treatment, compared to at diagnosis (Fig. 4b).

### Correlation between K/T ratio and clinical variables

**Fig. 5:**
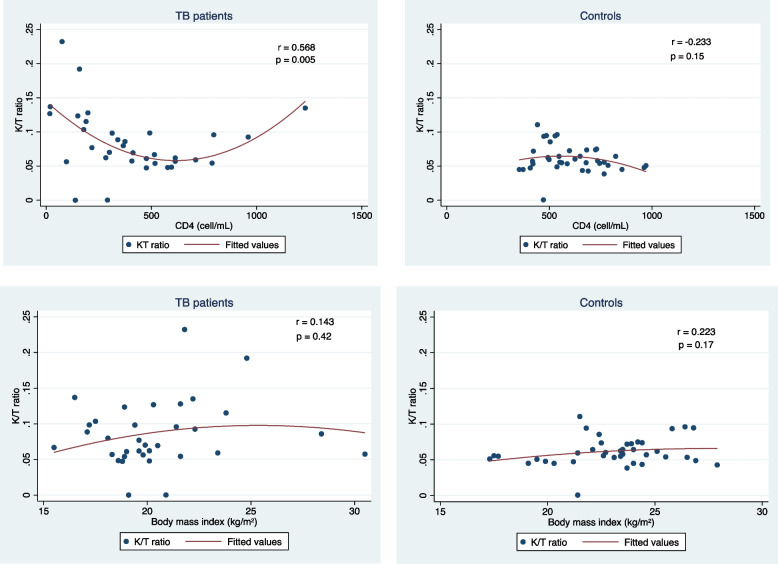
Plasma K/T ratios showed a quadratic association with CD4 cell counts in participants with TB disease, but not in controls. Body mass index showed no correlation with K/T ratio in either group

We analyzed the correlation of plasma K/T ratios with clinical variables of TB patients and controls. Plasma K/T ratios showed a parabolic correlation with CD4 cell counts in participants with TB (*p* = 0.005), but there was no correlation in controls (Fig. 5). There was no correlation between plasma K/T ratio and BMI in either group.

### Diagnostic value of plasma K/T ratio for tuberculosis among PWH

**Fig. 6:**
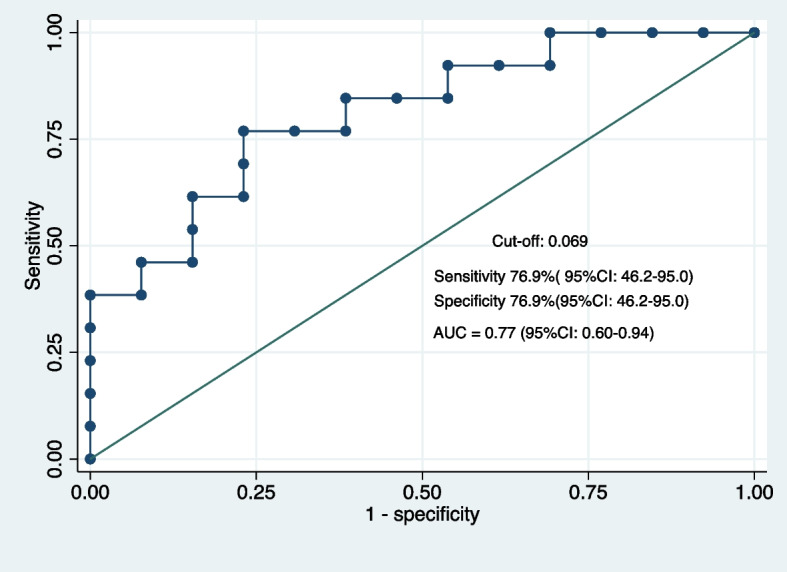
Diagnostic performance of plasma K/T ratio for tuberculosis diagnosis in PWH

We investigated the performance of plasma K/T ratio to diagnose TB disease in PWH, using both bacteriologically confirmed and clinically diagnosed TB at diagnosis as true-positives. For controls, we used the K/T ratio at the corresponding visit as true-negatives. We found that the K/T ratio achieved an AUC of 0.77 (95%CI 0.60–0.94) for diagnosis of tuberculosis (Fig. 6).

According to the WHO target product profile (TPP) for a triage test to identify people suspected of having TB, the minimum requirements for sensitivity and specificity are 90% and 70%, respectively [16]. At a cutoff of 0.054, the K/T ratio provided a sensitivity of 92% and a specificity of 46%. At a cutoff of 0.064, the K/T ratio provided a specificity of 70% and sensitivity of 77%. The minimum WHO TPP requirements for a non-sputum based diagnostic test are 65% for sensitivity and 98% for specificity. At a cutoff of 0.070, the K/T ratio provided a sensitivity of 69% and a specificity of 77%. At a cutoff of 0.098, the K/T ratio provided a specificity of 100% against a sensitivity of 38%. 

## Discussion

Non-sputum-based biomarkers are needed to diagnose and predict the occurrence of TB disease and monitor the effect of TB treatment in order to reduce TB morbidity and mortality in PWH. In this study, we assessed IDO activity by measuring plasma K/T ratios to diagnose and predict TB disease and monitor the response to TB treatment in PWH on ART from a longitudinal HIV cohort. We found that baseline plasma K/T ratios did not predict the occurrence of TB disease over a 6-month period, but were significantly higher among PWH with TB disease compared to PWH without TB at the time of TB diagnosis. In addition, the plasma K/T ratio significantly declined at 6 months relative to the time of TB diagnosis among TB patients who responded to treatment.

These findings are consistent with previous studies from an African TB cohort with HIV co-infection [15] and a population of HIV-negative persons with pulmonary TB disease from Georgia [13]. However, unlike the African TB cohort with HIV [15], we found no significant differences between the two groups from our study at 6 months before TB diagnosis. A possible explanation for this difference is that the plasma K/T ratios in our study were 15.7- fold lower compared to those of the PWH with TB in the African cohort (0.086 *vs* 1.35). It is possible that this difference is due to the use and duration of ART. Our participants were on long-term ART with a median duration of 12.2 years, whereas only 50% of the participants in the African cohort received ART at the time of TB diagnosis and were followed up to only 4 years. Previous cohort studies among PWH showed significantly decreased K/T ratios after ART initiation [17–19]. However, these levels remained higher compared to those observed in individuals without HIV infection [18] and the elevated IDO level did not normalize, even after more than 7 years of ART [20].

During the course of HIV infection, elevated IDO activity leads to a distortion in the differentiation of CD4 T-cells and directly hampers T-cell immune responses, thus contributing to HIV disease progression [21]. An in vitro study showed that IDO mRNA expression is elevated in peripheral blood mononuclear cells (PBMCs) from PWH compared with healthy controls, and inhibition of IDO results in increased CD4 + T-cell proliferative responses in PBMCs from PWH [22]. A cohort study among PWH initiating ART demonstrated that a higher KT ratio predicted poor CD4 + T-cell count recovery and increased mortality [17]. IDO activity also positively correlates with the size of HIV DNA reservoir in PBMCs of PWH receiving ART [23]. In addition, the level of IDO activities in the PWH co-infected with TB are higher compared to those with HIV mono-infection, both before and after ART initiation [24].

The plasma K/T ratios at the time of TB diagnosis in our study were comparable to those in a TB cohort without HIV from Georgia and a multidrug-resistant TB cohort from South Africa of which 75% of participants were HIV positive [13].

We found a trend toward decreased plasma K/T ratios in successfully treated patients, in line with previous studies [12, 13, 15]. However, we also observed 3/10 successfully treated patients did not have any change in K/T ratio after treatment. It is possible these patients had other inflammatory or infectious conditions, as the kynurenine pathway can be activated by infectious agents, inflammatory mediators and stress [25]. The participants who experienced treatment failure had increased K/T ratios after treatment, paralleled by a decrease in CD4 counts. Thus, the K/T ratio may have potential as a biomarker for TB treatment monitoring [26]. Current recommendations for TB treatment monitoring rely on sputum and culture conversion. However, sputum smear has a low sensitivity and cannot identify viable bacilli. Also, *M. tuberculosis* culture has a long turnaround time and risk for cross-contamination. In addition, a study among HIV-negative pulmonary TB patients in Japan showed that higher IDO activity at the time of TB diagnosis was an independent predictor for mortality [14].

A remarkable finding from the detailed analyses of various kynurenine metabolites was that patients who developed TB disease had lower baseline values for kynurenic acid, xanthurenic acid and quinolinic acid at all time points (Fig. 2). Kynurenine can be metabolized via three pathways by the enzymes kynureninase (KYNU), kynurenine 3-monooxygenase (KMO) and kynurenine aminotransferase (KAT) (Supplementary Fig. 1). Kynurenine is predominantly metabolized by KMO [27]. From these findings, it appears that both the KYNU and KAT activity are reduced in TB patients, and/or the clearance of these metabolites is enhanced in this group. It is important to realize that the systemic levels of these kynurenine metabolites may not reflect local levels, and further research is needed to understand whether these differences contribute to developing TB disease, or were found by chance.

The K/T ratio showed a significant parabolic (U-shaped) correlation with CD4 cell counts in TB participants but not in the controls. In vitro studies reported that the tryptophan catabolites kynurenine and picolinic acid can inhibit T-cell proliferation [28] and in vitro inhibition of IDO activity in peripheral blood mononuclear cells from PWH resulted in increased CD4 T-cell counts [22]. A study among PWH in Uganda showed that a higher K/T ratio at 12 months after ART initiation could predict slower CD4 T-cell count recovery, after adjusting for pre-ART CD4 T-cell count, viral load, age, and sex [17]. This suggests the correlation between high K/T ratio and low CD4 T cell count may be related to the cytotoxic and/or proliferation inhibitory effects of kynurenine. We also found a correlation between high K/T ratio and high CD4 T cells count. Since CD4 T cells are key components of the immune response against *M. tuberculosis*, high CD4 cell counts can enhance responses to TB leading to more IDO activity. We found no correlation between K/T ratio and BMI in either of our study groups.

Our analysis showed that the plasma K/T ratios achieved acceptable accuracy for diagnosing TB disease (AUC = 0.77). At a cutoff of 0.054, the K/T ratio produced a sensitivity of 92% and a specificity of 46%, which does not meet the current minimum WHO TPP requirements for a triage test. However, triage tests need to be simple low-cost tests, that can be used by low-skilled providers to rule out TB at the point-of care. Therefore, to serve as a triage test, the K/T ratio will require technological development. A recent study evaluating the K/T ratio in plasma from PWH with TB disease compared to those without TB, using enzyme-linked immunosorbent assay (ELISA) based on monoclonal antibodies, showed good agreement against mass spectrometry [29]. We also showed that K/T ratio may have utility for TB treatment monitoring, although we could not compare its accuracy against agreed requirements as there is currently no WHO TPP for this use.

There were several potential limitations to our study. First, we had a small sample size that limits the precision of our results. Second, we did not include individuals with other lung diseases to compare with the TB patients. However, a previous study among PWH showed that the K/T ratio had discriminatory power to differentiate between TB and pneumonia patients [15]. Last, we did not determine the contribution of either of the enzymes, TDO, IDO1 and IDO2, that convert tryptophan to kynurenine. The contribution of TDO to systemic kynurenine and downstream metabolites is likely to be low, as TDO also functions in healthy individuals without leading to systemic kynurenine and downstream metabolites. We cannot exclude a role for IDO2, which is less active than IDO1 and its level of expression is not known. Nevertheless, the kynurenine levels and levels of downstream metabolites were discriminatory, independent of which of the IDO isoforms contributed.

## Conclusions

In summary, our study demonstrated the plasma K/T ratio, measured by UPLC-MS/MS, had ability to identify TB disease and may serve as an adjunctive biomarker for TB treatment monitoring among PWH. Further studies conducted in well-designed prospective cohorts are necessary to validate our study results, and ascertain the optimal plasma K/T ratio cut-off for diagnosis of TB disease in PWH.

### Supplementary Information

**Supplementary Material 1.**

## Data Availability

The datasets generated and/or analysed during the current study are available from the corresponding author upon reasonable request.

## Abbreviations

ALT: Alanine aminotransferase
ART: Antiretroviral therapy
BMI: Body mass index
CI: Confidence interval
EPTB: Extra-pulmonary TB
EDTA: Ethylene diamine tetra acetate
IDO1: Indoleamine 2, 3-dioxygenase-1
IQR: Interquartile range
MRM: Multiple reaction monitoring
Kyn: Kynurenine
KYNU: Kynureninase
KAT: Kynurenine aminotransferase
KMO: Kynurenine 3-monooxygenase
K/T ratio: Kynurenine/ tryptophan ratio
PWH: People living with HIV
ROC: Receiver operating characteristic curve
Sum/T ratio: Sum of all determined kynurenine metabolites to tryptophan concentration
Trp: Tryptophan
TB: Tuberculosis
UPLC-MS/MS: Ultra-performance liquid chromatography, a tandem mass spectrometer
