# Supplementary material for: The plasma kynurenine-to-tryptophan ratio as a biomarker of tuberculosis disease in people living with HIV on antiretroviral therapy: an exploratory nested case–control study
Source: BMC Infect Dis. 2024 Apr 2;24:372. doi: 10.1186/s12879-024-09258-4 (PMC10988902; doi:10.1186/s12879-024-09258-4)
Supplement: Supplementary file 1 — Supplementary Material 1. [file 12879_2024_9258_MOESM1_ESM.pdf]

Supplementary Appendix

This appendix has been provided by the authors to give readers additional information about their work.

Supplement to: Gatechompol S, Lutter R, Vaz FM, et al. The plasma kynurenine-to-tryptophan ratio as a biomarker of tuberculosis disease in people living with HIV on antiretroviral therapy: An exploratory nested case-control study

Figure S1. Tryptophan and downstream metabolites pathway.

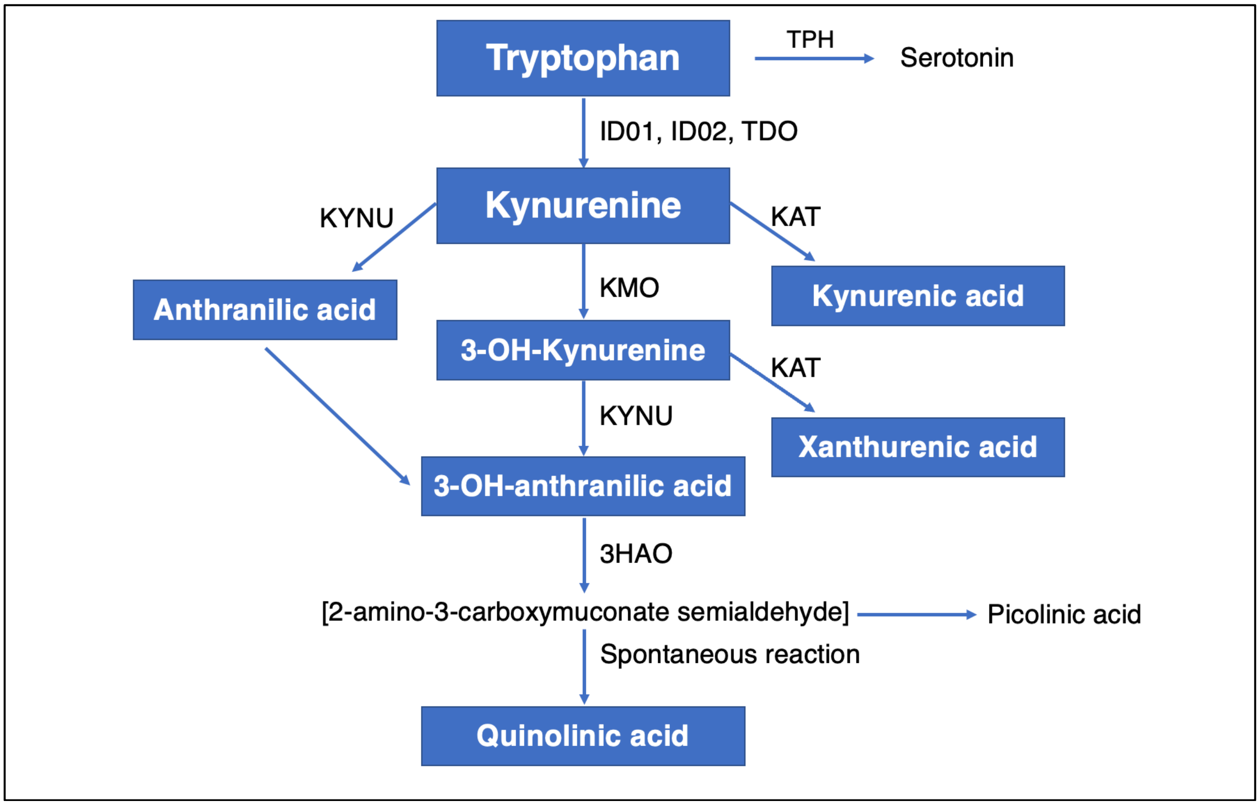

Abbreviations: IDO, indoleamine 2,3-dioxy genase; TDO, tryptophan 2,3-dioxygenase; TPH, tryptophan hydroxylase; KAT, kynurenine aminotransferase; KYNU, kynureninase; KMO, kynurenine 3-monooxygenase; 3HAO, 3-hydroxyanthranilate 3,4-dioxygenase.

Figure S2. A sensitivity analysis of K/T ratio comparing participants with TB treated successfully and controls

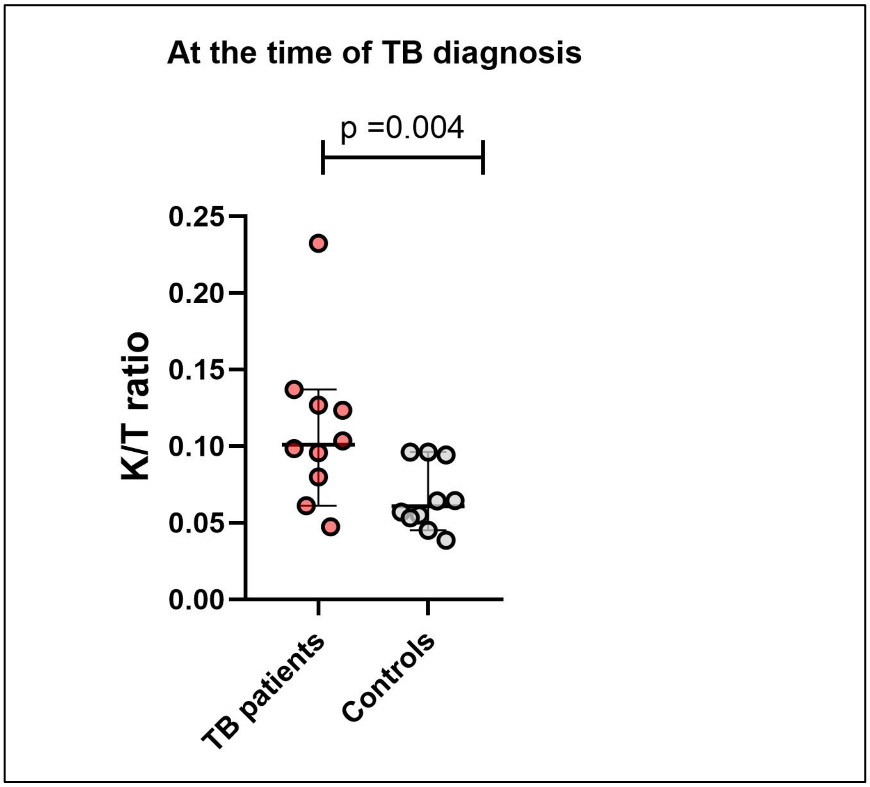

*The p value was calculated using Mann-Whitney test
Abbreviations: TB: tuberculosis; Dx: Diagnosis; K/T ratio: kynurenine/ tryptophan ratio
